# The complete chloroplast and mitochondrial genomes of *Scopelophila cataractae* (Mitt.) Broth. (Pottiaceae, Bryophyta)

**DOI:** 10.1080/23802359.2021.2013742

**Published:** 2021-12-29

**Authors:** Yuya Inoue, Miho Nakahara-Tsubota, Hiromi Tsubota

**Affiliations:** a Department of Botany, National Museum of Nature and Science, Ibaraki, Japan; b Hattori Botanical Laboratory, Miyazaki, Japan; c Natural History Museum and Institute, Chiba, Japan; d Miyajima Natural Botanical Garden, Graduate School of Integrated Sciences for Life, Hiroshima University, Hiroshima, Japan

**Keywords:** Bryophyta, chloroplast genome, copper moss, mitochondrial genome, Merceyoideae, organelle genome, phylogenetic relationships, Pottiaceae

## Abstract

The complete chloroplast and mitochondrial genome sequences of *Scopelophila cataractae* (Pottiaceae, Bryophyta) are determined. The chloroplast genome is 122,290 bp with 118 genes and the mitochondrial genome is 105,607 bp with 67 genes, both genomes are circular. This study showed the *S. cataractae* plastome contains the smallest genome size, and a functional *trnP*^GGG^ gene, relative to other pottiaceous species. Phylogenetic inferences support the sister relationship of *S. cataractae* to all other pottiaceous accessions.

Pottiaceae is one of the richest families of Bryophyta, comprising more than 1,400 species in approximately 80 genera (Zander 1993). *Scopelophila cataractae* (Mitt.) Broth. is well known as a ‘copper moss’ that can survive in copper-rich environments, such as around temples and shrines with copper roofs, bronze artifacts, abandoned mines, and smelters (Satake et al. 1988; Satake 2014). The species accumulate copper in the cell wall pectin (Konno et al. 2010), although the molecular mechanisms of accumulation have not been revealed. In vascular plants, metal tolerance and homeostasis are maintained by chelating, effluxing, or sequestering molecules (Clemens 2001; Hall 2002). In addition to the unique ecological features of *S. cataractae*, molecular phylogenetic studies have revealed that it was included in the basalmost clade within the Pottiaceae (Werner et al. 2004; Cox et al. 2010; Inoue and Tsubota 2016), corresponding to the subfamily Merceyoideae. The genomic resources of organelle have been provided in several genera of the Pottiaceae (*Chionoloma*: Alonso et al. 2016, as *Oxystegus*; *Pseudocrossidium*: Cevallos et al. 2019, 2020; *Syntrichia*: Oliver et al. 2010; Yoon et al. 2016; Kim et al. 2019). The genomic data are however still limited in the family and lack in the major lineages. Here we present the chloroplast (cp) and mitochondrial (mt) genomes of *S. cataractae* as the resources for a better understanding of genomic structure and evolutions within Pottiaceae. The phylogenetic relationships within the family were also inferred based on the protein-coding sequences of cp and mt genomes.

Samples of *S. cataractae* were collected from Tochigi Prefecture, Japan (36°46′16″N 139°42′41″E). A specimen was deposited at the Herbarium of Hiroshima University (HIRO; Director: Tomio Yamaguchi, yamatom@hiroshima-u.ac.jp) under the voucher number *Y. Inoue 4216*. The total DNA was extracted from the axenic *in vitro* culture of protonemal filaments derived from a single spore (Takio 1995, modified), with NucleoSpin Plant II (Macherey–Nagel, Duren) following the manufacturer’s protocols, and sequenced using the Illumina MiSeq platform. A total of approximately 693 K raw reads was analyzed, comprising an average fragment length of 150 bp. Low-quality reads (<Q30), abnormal short reads (<20 bp), and adapter sequences were trimmed using fastp 0.20.0 (Chen et al. 2018). After quality control, the GetOrganelle 1.7.1 (Jin et al. 2020) was used to assemble the filtered reads with the seed reads comprising published organelle genome sequences of mosses, and the assembled sequences were polished by Pilon 1.23 (Walker et al. 2014). The polished sequences were annotated using GeSeq 2.03 (Tillich et al. 2017) and manually corrected using the SnapGene 5.2.3 (from GSL Biotech; snapgene.com). Since the chloroplast sequence still contained one gap, the specific primers were designed to bridge the gap with PCR amplification and conventional Sanger sequencing by ABI 3730xl. The final annotated cp and mt sequences were submitted to the DNA Data Bank of Japan (DDBJ) and assigned accession numbers LC634773 for cp and LC634774 for mt.

Phylogenetic analyses were conducted with protein-coding sequences of chloroplast (79 genes) and mitochondrial (38 genes) genomes, respectively. Each data matrix consists of representative species selected from major lineages of mosses based on Liu et al. (2019), including all pottiaceous accessions. Sequences were aligned using MAFFT 7.475 (Katoh and Standley 2013), with some manual adjustments by the sequence editor of MEGA 7.0.26 (Kumar et al. 2016). Start and stop codons were removed, and gaps were treated as missing data. Kakusan4 (4.0.2016.11.07; Tanabe 2011) was used to determine the appropriate substitution model and partitioning scheme for our data based on the corrected Akaike information criterion (AICc: Sugiura 1978). RAxML 8.2.9 (Stamatakis 2014) was used for maximum likelihood inference using GTR + Γ model, with a rapid bootstrap analysis of 1,000 replicates.

*Scopelophila cataractae* had a 122,290 bp circular chloroplast genome, which is the smallest among the published cp genomes of the Pottiaceae. The cp genome had a GC content of 28.05% and a typical quadripartite structure, consisting of a large single-copy (LSC) region of 83,728 bp, a small single-copy (SSC) region of 18,620 bp, and a pair of inverted repeats (IRs) of 9,971 bp. It contained 118 genes, including 82 protein-coding genes, 32 tRNA genes, and four rRNA genes. Among the published cp genomes of the Pottiaceae, *trnP*^GGG^ gene is absent or pseudogenized, while in the *S. cataractae*, this gene appears to be functional according to the tRNA structure prediction by tRNAscan-SE 2.0 (Lowe and Chan 2016). The mitochondrial genome was circular, with 105,607 bp, and a GC content of 39.11%. It contained 67 genes, including 40 protein-coding genes, 24 tRNAs, and three rRNAs.

Both cp and mt trees strongly supported the sister relationship of *S. cataractae* to all other pottiaceous accessions (Figure 1). This result is consistent with previous phylogenies based on the selected cp genes (Werner et al. 2004; Cox et al. 2010; Inoue and Tsubota 2016).

**Figure 1.**
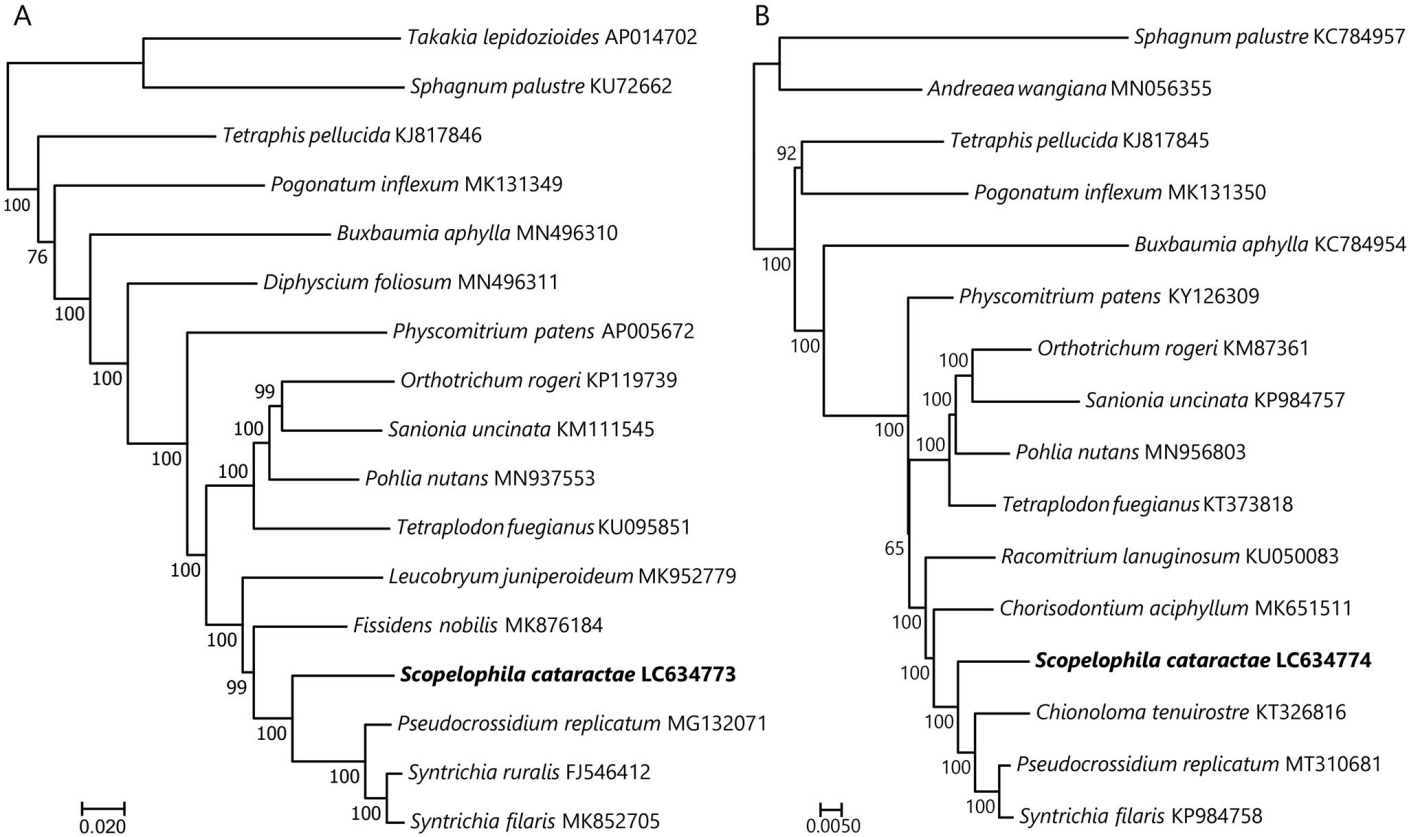
Maximum-likelihood trees of mosses inferred from the 79 chloroplast (A) and 38 mitochondrial (B) protein-coding sequences. Bootstrap values of 1,000 replicates by RAxML are shown on the branches. The root is arbitrarily placed on the branch leading to the clades that include members of the genera *Sphagnum*, and *Takakia* or *Andreaea*.

## Data Availability

The genome sequence data that support the findings of this study are openly available in DDBJ (https://www.ddbj.nig.ac.jp/) and NCBI (https://www.ncbi.nlm.nih.gov/), under the accession numbers LC634773 and LC634774. The associated BioProject, BioSample, and SRA numbers are PRJDB11883, SAMD00334531, and DRR303909 respectively.
